# The heroes among us: Leveraging Africa’s youth for effective digital health implementation in communities

**DOI:** 10.1371/journal.pdig.0000954

**Published:** 2025-07-24

**Authors:** Esther Opone

**Affiliations:** University of Cape Town, Cape Town, South Africa; McGill University, CANADA

*Digital technology will not solve Africa’s healthcare challenges…*

This might sound ironic, especially coming from an African digital health professional. However, it is informed by practical experience implementing digital health solutions in diverse contexts, as well as evidence from literature highlighting the persistent gaps between technological innovation and successful adoption in low-resource settings.

It is well established that digital innovation has the great potential to improve healthcare, including bridging access gaps through telemedicine, improving health literacy through digital communication and boosting chronic disease management with e-health tools [1]. However, realizing that potential transcends having the best technology alone, it requires effective implementation and change management strategies, particularly in addressing the complex human factors that threaten successful and sustained adoption [2]. This challenge is especially critical in Africa’s underserved settings, where there seems to be more cultural barriers and skepticism towards new technologies, which greatly shape user engagement [3]. Without proper change management to address these issues and improve adoption, the potential benefits of digital health may not fully materialize [4].

While various efforts have been made to tackle implementation challenges at the community level, this opinion piece seeks to highlight a powerful (yet often-overlooked/relegated) pool of heroes capable of helping to navigate these dynamics—African youths.

## Why the youth?

With over 400 million youths, Africa has the youngest population globally [5]. This, alongside increasing mobile phone penetration and internet usage, makes the continent fertile ground for digital innovations to thrive, including digital health [6,7]. Asides their demographic power, fresh perspectives, creativity, and resilience, many other factors uniquely position African youths as not just contributors (often brought in as an after-thought), but pivotal leaders and drivers of community-centered digital health change management efforts. These include:

- **Tech-Savviness and Influence:** Youths are likely to adopt new technologies faster than their older counterparts and influence others [8]. This makes them key change agents who can be leveraged to introduce and normalize digital health innovations within their communities. Where skepticism and distrust exist, they can help overcome user resistance by advocating, teaching and demonstrating the benefits of digital health tools to others. Studies show that these enhance digital health literacy, which increases chances of successful adoption and positive outcomes [7,9].
- **Co-Design, Representation, and Feedback:** With strong ‘insider’ understanding of local dynamics, Africa’s youths can serve as intermediaries between their communities and digital health creators and/or implementers. For instance, by easing community entry, stakeholder engagement process, and facilitating real-time feedback, they can ensure that digital health initiatives are culturally-sensitive, contextually-relevant, user-friendly, and effective in meeting the needs of their communities. These ultimately contribute to more effective implementation, such as seen in youth engagement in the co-design of CyberRwanda in Rwanda [10].
- **Sustainability:** Beyond the initial uptake, a community’s youths can be key drivers in embedding digital health solutions into their local health ecosystem and ensuring their long-term success. This works especially when they adopt a sense of ownership and foster the same among their peers and within their communities. The implementation of TuneMe, a digital intervention for sexual and reproductive health and rights (SRHR) in Zambia, is an example of this [11].

## Recommendations

Leveraging Africa’s youth for effective digital health implementation in communities must go beyond mere tokenism. Digital health implementers, donors, and policymakers must recognize youths in target communities as relevant stakeholders and “honor” their digital health citizenship [12]. To this end, the following recommendations are proposed:

1 **Holistic Youth Capacity-Building for Digital Health Implementation:** While many African countries are investing in digital literacy among youths to meet the demands of an increasingly digital world, effective digital health implementation requires holistic capacity-building beyond general platform skills. This includes training in community mobilization, digital change management, health data ethics, and understanding of local health systems, to better prepare youths to serve as implementation liaisons and local sustainability champions. National health ministries and implementing partners should establish structured youth-focused initiatives that recruit and train youths and embed them within local digital health implementation teams. To navigate resource and logistical constraints, strategic partnerships with networks like Transform Health (https://transformhealthcoalition.org) and Digital Health Africa (https://digitalhealth-africa.org) can be forged. Existing models, such as Nigeria’s National Youth Service Corps (NYSC) Health Initiative for Rural Dwellers (HIRD), can also be adapted. For instance, the deployed medical corps members, who are already embedded and carrying out successful health promotion activities in local communities, can be further trained to support digital health implementation efforts [13].
2 **Institutionalize Youth Co-Design in Digital Health Initiatives:** Governments and implementers must formally include young people in the design and local adaptation of digital health tools, especially for services targeting adolescents and young adults, and/or communities that they are representatives of. This means not just piloting with youths, but embedding them in initial research, design, and decision-making cycles, as recommended in the WHO Digital Health Youth Framework [11]. This ensures proper community participation and protection, especially in vulnerable African communities.
3 **Integrate Youth Representation in Digital Health Governance Structures:** Beyond the direct digital tool or platform, it is important to include youths in relevant policy and implementation discussions, such as in national digital health steering committees, policy advisory groups, and regulatory bodies. This ensures that the needs and valuable insights of this majority demographic are not overlooked in strategic decisions as digital innovations in health continue to evolve.

## Conclusion

This article has emphasized that digital technology alone will not solve Africa’s healthcare challenges. Rather, its impact depends on how well it is adopted, adapted, and sustained within communities. By positioning youths not as afterthoughts but as central actors in effective implementation and change management, we can harness their potential as catalysts for sustainable digital health solutions in communities. This requires deliberate investments in youth capacity-building, institutionalized co-design, and inclusive governance structures. Only then can we move closer to seeing digital technology effectively and sustainably solve Africa’s healthcare challenges.

## References

[1] Stoumpos AI, Kitsios F, Talias MA. Digital transformation in healthcare: technology acceptance and its applications. Int J Environ Res Public Health. 2023;20(4):3407. doi: 10.3390/ijerph20043407 36834105 PMC9963556

[2] Schueller SM. Grand challenges in human factors and digital health. Front Digit Health. 2021;3:635112. doi: 10.3389/fdgth.2021.635112 34713105 PMC8522014

[3] Ade-Ibijola A, Okonkwo C. Artificial intelligence in Africa: emerging challenges. In: Responsible AI in Africa: challenges and opportunities. Cham: Springer International Publishing; 2023. p. 101–17.

[4] Ezeudoka BC, Fan M. Exploring the impact of digital distrust on user resistance to e-health services among older adults: the moderating effect of anticipated regret. Human Soc Sci Commun. 2024;11(1):1–19.

[5] Statista. Proportion of selected age groups of the world population in 2024, by region. 2025. Available from: https://www.statista.com/statistics/265759/world-population-by-age-and-region/

[6] GSMA. The mobile economy 2023. 2023. Available from: https://www.gsma.com/solutions-and-impact/connectivity-for-good/mobile-economy/wp-content/uploads/2023/03/270223-The-Mobile-Economy-2023.pdf

[7] Abdul Hamid Alhassan RH, Haggerty CL, Fapohunda A, Affan NJ, Anto-Ocrah M. Exploring the use of digital educational tools for sexual and reproductive health in sub-Saharan Africa: systematic review. JMIR Public Health Surveill. 2025;11:e63309. doi: 10.2196/63309 40009849 PMC11904370

[8] Wong BLH, Holly L, Gray W, van Kessel R. Youth: Key drivers of digital adoption and health data governance. TEN. 2021;27(2):18.

[9] Fitzpatrick PJ. Improving health literacy using the power of digital communications to achieve better health outcomes for patients and practitioners. Front Digit Health. 2023;5:1264780. doi: 10.3389/fdgth.2023.1264780 38046643 PMC10693297

[10] Nolan C, Packel L, Hope R, Levine J, Baringer L, Gatare E, et al. Design and impact evaluation of a digital reproductive health program in Rwanda using a cluster randomized design: study protocol. BMC Public Health. 2020;20(1):1701. doi: 10.1186/s12889-020-09746-7 33187485 PMC7662730

[11] World Health Organization. Youth-centered digital health interventions: a framework for planning, developing and implementing solutions with and for young people. World Health Organization; 2020.

[12] Digital Transformations for Health Lab. Brief: digital health citizenship. Geneva: DTH-Lab; 2024. Available from: https://dthlab.org/wp-content/uploads/2024/05/2024_Digital-Health-Citizenship.pdf

[13] Premium Times. Four million rural dwellers benefitted from NYSC’s health initiative in 11 years-DG: Premium Times. 2025. Available from: https://www.premiumtimesng.com/news/top-news/795486-four-million-rural-dwellers-benefitted-from-nyscs-health-initiative-in-11-years-dg.html?tztc=1

